# Integrative Multi-Omics Reveals the Anti-Colitis Mechanisms of *Polygonatum kingianum* Collett & Hemsl Polysaccharides in a Mouse DSS Model

**DOI:** 10.3390/nu17172895

**Published:** 2025-09-08

**Authors:** Siyu Li, Xingrui Xu, Yuezhi Pan, Yu Chen, Zihuan Wu, Shengbao Cai

**Affiliations:** 1 Faculty of Food Science and Engineering, Kunming University of Science and Technology, Kunming 650500, China; lsy.913@foxmail.com (S.L.); xuxingruiangle@163.com (X.X.); 202311409120@stu.kust.edu.cn (Y.P.); kustchenyu2023@gmail.com (Y.C.); 2 Yunnan Specialty Food Biosynthesis and Biomanufacturing Group, Kunming University of Science and Technology, Kunming 650500, China; 3 State Key Laboratory of Food Science and Resources, Jiangnan University, Wuxi 650500, China

**Keywords:** *Polygonatum kingianum* polysaccharides, DSS-induced colitis, intestinal barrier, multi-omics integration

## Abstract

**Background/Objectives**: Ulcerative colitis (UC) incidence has risen alarmingly worldwide, posing significant clinical challenges due to limitations of therapeutic efficacy and side effects of current drugs. While *Polygonatum kingianum* polysaccharides (PKPs) exhibit anti-inflammatory and antioxidant properties, their anti-colitis potential remains unexplored. This study aimed to validate the protective effects of PKPs against dextran sulfate sodium (DSS)-induced colitis and elucidate its mechanisms. **Methods**: Acute UC was induced in C57BL/6J mice by 3% DSS. PKPs (125 mg/kg) were administered via gavage for 10 days. Integrated approaches included histopathology, tight junction protein (ZO-1/Occludin/Claudin-1) immunohistochemistry, inflammatory/oxidative markers (ELISA), Nrf2 pathway proteins (Western blot), 16S rRNA gut microbiota sequencing, fecal untargeted metabolomics (UHPLC-MS), short-chain fatty acids (SCFAs) analysis and combined analysis. **Results**: PKPs significantly alleviated colitis phenotypes: reduced weight loss, lowered disease activity index (DAI), and attenuated colon shortening. They restored intestinal barrier integrity by upregulating tight junction proteins and reducing plasma Diamine Oxidase (DAO)/D-lactate (D-Lac)/Endotoxin (ET). PKPs suppressed pro-inflammatory cytokines (TNF-α/IL-1β/IL-6) while elevating IL-10, activated the Nrf2/HO-1/NQO1 antioxidant pathway, and reduced oxidative stress (MDA decreased, SOD/GSH increased). Multi-omics revealed PKPs enriched beneficial bacteria (*Blautia*, *Odoribacter*, *Rikenellaceae_RC9_gut_group*), restored SCFAs (acetate/propionate/butyrate), and modulated metabolic pathways (sphingolipid/linoleic acid metabolism). **Conclusions**: PKPs ameliorate DSS-induced colitis through multi-target mechanisms: (1) preserving intestinal barrier function, (2) suppressing inflammation and oxidative stress via Nrf2 activation, (3) restoring gut microbiota balance and SCFA production, and (4) regulating host-microbiota metabolic interactions. These findings support PKPs as a promising dietary supplement for UC management.

## 1. Introduction

Over the past several decades, the global incidence of inflammatory bowel disease (IBD) has increased steadily, particularly in newly industrialized nations [1]. Specifically, the incidence of IBD in China has alarmingly nearly tripled in recent years [1]. During the disease course, approximately half of IBD patients require hospitalization, while 10–20% ultimately undergo surgical intervention [1,2]. IBD, which includes Crohn’s disease and ulcerative colitis (UC), is a group of chronic immune-mediated disorders. UC specifically involves persistent mucosal inflammation, ulceration, and bleeding limited to the colon [3,4]. The pathogenesis of UC involves multifactorial interactions, including genetic susceptibility, immune dysregulation, gut microbiota perturbations, and environmental triggers. Beyond substantially impairing patients’ quality of life, progressive UC confers an elevated risk of colorectal cancer [2]. Despite the availability of contemporary therapeutic strategies including 5-aminosalicylic acid derivatives, thiopurines, biologic agents, and small-molecule drugs, clinical management continues to face significant challenges due to adverse effects, variable treatment responses, and drug resistance [5]. To address these situations, auxiliary treatment is currently proposed. Auxiliary treatment mainly includes alternative therapies, nutritional support, and some other methods [2,5]. Alternative therapies mainly use agents such as plant extracts, specific nutrients, and probiotics as auxiliary treatment. The main purposes are to relieve symptoms, reduce recurrence rate, and improve patients’ quality of life [2,5].

Polysaccharides, characterized by structural diversity, low toxicity, and broad-spectrum bioactivity, demonstrate significant potential for applications in healthcare and food industries [6]. In recent years, polysaccharides have garnered substantial interest for their therapeutic roles in inflammatory diseases. Accumulating evidence indicates that polysaccharides from diverse sources exhibit multifaceted biological properties, including anti-inflammatory, antioxidant, and microbiota-modulating effects [7]. Notably, polysaccharides derived from Gracilaria lemaneiformis, tea, and oysters have demonstrated efficacy in ulcerative colitis (UC) management [8,9,10]. *Polygonatum kingianum* Collett & Hemsl (*P. kingianum*), a revered traditional Chinese tonic, has been historically employed to invigorate splenic function, promote digestion, and suppress cough. *P. kingianum* polysaccharides (PKPs) constitute primary bioactive constituents of this herb. Recent investigations confirm that PKPs possess marked biological activities, particularly anti-inflammatory properties and oxidative stress inhibition [11,12].

Based on the discussion above, we speculate that PKPs may be a candidate polysaccharide for alleviating UC. The primary objective of this paper is to investigate whether it possesses colitis-alleviating properties and elucidate the underlying mechanisms. We employed DSS-induced colitis models to simulate the disease progression observed in humans. Given that colitis pathogenesis involves a complex network of multidimensional interactions—including immune imbalance, intestinal barrier damage, microbiota dysbiosis, and metabolic dysregulation [2]—conventional single-indicator research methods are inadequate for comprehensively revealing whether PKPs exert protective effects against colitis. Therefore, this study integrates animal models, histopathological analysis of colon tissues, and multi-omics technologies to validate this hypothesis.

## 2. Methods and Materials

### 2.1. Materials and Reagents

The Diamine Oxidase (DAO), D-lactate (D-Lac), Myeloperoxidase (MPO), Superoxide Dismutase (SOD), Malondialdehyde (MDA), Catalase (CAT) and Glutathione (GSH) kits were obtained from Nanjing Jiancheng Bioengineering Institute (Nanjing, China). The Endotoxin (ET), TNF-α, IL-1β, IL-6, and IL-10 kits were purchased from Shanghai Enzyme-linked Biotechnology (Shanghai, China). Antibodies targeting GAPDH, NRF2, HO-1, and NQO1 were obtained from Affinity Biosciences (Changzhou, China).

### 2.2. Preparation of PKPs

The PKPs used in this study were extracted and characterized previously in our laboratory. The number-average molecular weight (Mn), weight-average molecular weight (Mw), and polydispersity index (PD) of PKPs were 4981 Da, 13,578 Da, and 2.73, respectively, with molecular weights primarily distributed in the ranges of 1749–5000 Da (41.9%) and 5000–10,000 Da (52.0%). The monosaccharide composition consisted of glucose (Glc) and fructose (Fru), with a molar ratio of 0.071:0.929. More detailed information is available in our previous publication [13].

### 2.3. DSS-Induced Acute UC Mouse Model

The animal experiment process was carried out in accordance with the laboratory animal ethics and usage guidelines implemented by the National Institutes of Health of the United States. The use of animals was approved by the Institutional Animal Ethics Committee (IAEC) of Kunming University of Science and Technology (Approval Number: PZWH-KUST-202501020019-9)

**Figure 1:**
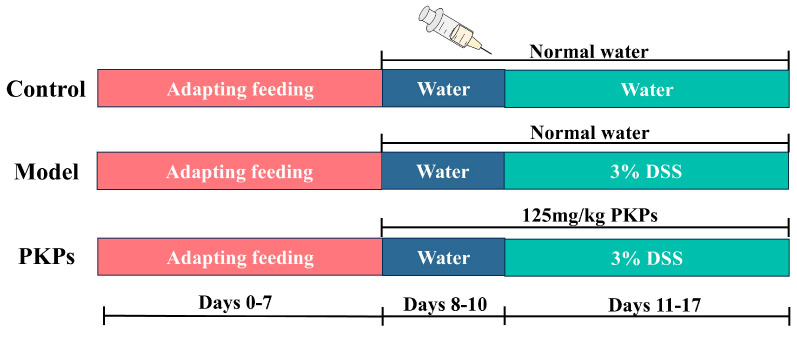
Roadmap of Experimental Techniques for DSS-Induced Acute UC in Mice.

7-week-old male C57BL/6J mice weighing 20 ± 2.0 g were used in the experiments, purchased from Hunan Silaike Jingda Experimental Animal Co., Ltd. (SCXK (Xiang) 2021-0002, Changsha, China). The animal were maintained on a free-access diet and sterile water under standard environmental conditions (23 °C ± 2 °C, 40–75% humidity). After adapting for 7 days, mice were randomly assigned to three groups (PKPs, Control, and Model, eight animals per group, 24 in total) using a random number table generated by IBM SPSS 27 software. All mice were housed under specific pathogen–free (SPF) conditions, and animals within the same group were co-housed (4 mice per cage, two cages per group). Figure 1 illustrates the DSS-induced UC animal model and the treatment schedule with PKPs. Based on pre-experiments and previous studies, an appropriate dose of PKPs was determined [14]. After the experiment began, mice were administered *P. kingianum* polysaccharide at a dose of 125 mg/kg body weight (oral gavage volume: 10 mL/kg) via oral gavage daily from Day 8 to Day 17. Distilled water was administered to both the Control and Model groups as a blank treatment. The PKPs group received the corresponding dose of PKPs (dissolved in distilled water) via gavage. From Day 11 to Day 17, the mice were given drinking water that included DSS, which was made as a 3% (*w/v*) solution in distilled water and filtered through a 0.22 μm filter. The Control group received normal distilled water.

### 2.4. Macroscopic Evaluation Parameters

Macroscopic evaluation in this study comprised three key parameters: disease activity index (DAI), colon length, and spleen-to-body weight ratio. Throughout DSS induction, the mice’s body weight was measured daily, and fecal consistency and bleeding were monitored. The DAI score was determined based on the criteria specified in Table S1 [15]. On the seventh day, blood samples were collected by enucleation of the mice, followed by euthanasia via cervical dislocation.

### 2.5. Histopathological Scoring

Colon tissues were fixed, paraffin-embedded, sectioned, and stained with H&E for microscopic examination; detailed histological scoring criteria are provided in Table S2 [16].

### 2.6. Immunohistochemistry

Comprehensive experimental details are provided in Supporting Information S1. Positive areas were quantitatively analyzed using ImageJ 1.54f software after microscopic image acquisition [17,18].

### 2.7. Colon Barrier Evaluation

After blood collection from the eyeball, plasma was obtained by centrifugation, and the levels of ET, D-Lac, and DAO in the plasma were measured.

### 2.8. Inflammatory Cytokine and Oxidative Stress Measurement

A suitable amount of colon tissue was homogenized with 1:9 (*w*/*v*) saline, and the resulting 10% tissue homogenate was subjected to centrifugation. The supernatant was harvested, and the concentrations of MPO, SOD, MDA, CAT, GSH and TNF-α, IL-1β, IL-6, IL-10 were measured using commercial kits according to the manufacturer’s instructions.

### 2.9. Western Blot

Comprehensive experimental details are provided in Supporting Information S2. The densitometry of protein bands was analyzed with ImageJ software (National Institutes of Health) [19].

### 2.10. 16S rRNA Gut Microbiota Sequencing

During tissue collection, the cecal and colonic contents of mice were placed into sterile cryovials, snap-frozen in liquid nitrogen, and stored at −80 °C until analysis. DNA was isolated from the gut contents using the TIANamp Soil DNA Kit(TIANGEN Biotech (Beijing) Co., Ltd., Beijing, China). The V3–V4 region of the 16S rRNA gene was amplified via PCR with primers 341F (CCTAYGGGRBGCASCAG) and 806R (GGACTACNNGGGTATCTAAT).Following PCR product purification, libraries were constructed, quantified using Qubit and qPCR, and qualified libraries were sequenced on the Novaseq 6000 platform (Illumina, San Diego, CA, USA) using a PE250 strategy. All Effective Tags from all samples were clustered using the Uparse algorithm, with sequences clustered into OTUs at a default identity threshold of 97%. These OTUs were then used to calculate the richness and diversity indices as well as the relative abundance of the gut bacterial community. Data analysis was performed on the Novaseq cloud platform (https://magic.novogene.com/).

Alpha-diversity indices were calculated on the cloud platform, and statistical significance was assessed with the Wilcoxon rank-sum test. Beta-diversity was evaluated by principal coordinates analysis (PCoA) based on the weighted UniFrac algorithm and non-metric multidimensional scaling (NMDS) based on the unweighted UniFrac algorithm. Differential taxonomic abundance was analyzed using the Wilcoxon rank-sum test. LEfSe analysis (LDA score threshold = 2) was applied to identify dominant taxa across groups.

### 2.11. Short-Chain Fatty Acids (SCFAs)

The method is a modification based on previous work. The extraction and analysis of SCFAs from mouse fecal samples were conducted following the protocols established in our previous study [13]. Briefly, 50 mg of mouse feces was mixed with saturated sodium chloride (400 μL) and 10% H_2_SO_4_ (20 μL), vortexed for 2 min, and extracted with ether (800 μL) followed by ultrasonic treatment at 4 °C for 5 min. After centrifugation (3400 g, 5 min), the ether layer was collected, dried with anhydrous sodium sulfate, filtered (0.22 μm), and stored in brown glass vials for analysis.

Short-chain fatty acids were quantified by GC-MS (HP-INNOWax column, 30 m × 0.25 mm × 0.25 μm, Agilent, Santa Clara, CA, USA). The column oven was held at 100 °C for 1 min, then ramped to 155 °C at 5 °C/min (12 min total). Mass spectrometry was performed in EI mode (70 eV, 35–500 *m*/*z*), with ion source at 230 °C, transfer line at 280 °C, and helium carrier gas at 1 mL/min

### 2.12. Fecal Untargeted Metabolomics

Mouse fecal samples (100 mg) were placed in grinding tubes with two steel beads and 500 μL of Methanol/acetonitrile/water in a 2:2:1 (*v/v/v*) ratio. Samples were homogenized at 50 Hz for 300 s, then subjected to ultrasonication in an ice bath for 30 min. After standing at −30 °C for 2 h. The samples were centrifuged (11,000 g, 20 min, 4 °C) and the resulting supernatant was membrane-filtered (0.22 μm) for subsequent analysis.

C18 column (HYPERSIL GOLD Vanquish 1.9 µM 100 × 2.1 mm, Thermo Fisher, Waltham, MA, USA) was used to separate the metabolites. The conditions of chromatographic separation were as follows: chromatography was conducted at 40 °C column temperature with 0.2 mL/min flow rate and 2 μL injections. (A) 0.1% formic acid in water and (B) acetonitrile made up the mobile phase. The gradient program was as follows: 0–2 min, 2% B; 2–12 min, 2% to 100% B; 12–18 min, 100% B; 18.01–20 min, 2% B. An Orbitrap Exploris 120 mass spectrometer was used for mass spectrometry analysis following UHPLC separation. Sample ionization was performed via electrospray ionization (ESI) in positive ion mode. The ionization conditions were as follows: Spray Voltage: 3.4 kV; Sheath Gas: 50 Arb; Aux Gas: 10 Arb; Ion Transfer Tube: 320 °C; Vaporizer Temp: 350 °C; RF Lens: 70%.

The LC–MS raw data acquired from Xcalibur were processed using Compound Discoverer 3.0 (Thermo Fisher Scientific, Waltham, MA, USA). Database searches were performed against mzCloud spectral library, mzVault, ChemSpider™, Human Metabolome Database (HMDB), Kyoto Encyclopedia of Genes and Genomes (KEGG), MassBank, and BioCyc. Following peak alignment and baseline correction, metabolite identification in mouse fecal samples was conducted. The identification workflow was adapted from a previously reported protocol [20], with minor modifications. Specifically, features lacking MS/MS fragmentation spectra, with ΔMass (ppm) > 5, or exhibiting CV (%) ≤ 10% were excluded. The remaining features were subjected to MS/MS spectral interpretation and subsequently matched against multiple databases to enhance the reliability and accuracy of metabolite annotation. Differential analysis in Compound Discoverer was based on two-sample Student’s *t*-tests to compute *p*-values, with adjusted *p*-values provided to account for multiple testing. Differential metabolites were screened using mzCloud best match score > 70, log2 fold change ≥ 1 or ≤ −1, and *p*-value < 0.05 as the filtering criteria [20], combined with manual correction. Enrichment analysis of differential metabolites was conducted using the KEGG database implemented in MetaboAnalyst 4.0.

### 2.13. Data Analysis

Data are expressed as the mean ± SE. Except for 16S rRNA and untargeted metabolomics data, all analyses of statistical significance were conducted using IBM SPSS Statistics 27 software (SPSS, Chicago, IL, USA). Normality (Shapiro–Wilk test) and homogeneity of variances (Levene’s test) were first assessed. For data that followed a normal distribution with equal variances, one-way ANOVA was conducted, followed by Tukey’s test for multiple comparisons. Otherwise, Kruskal–Wallis test was used to determine significant differences. A *p*-value < 0.05 was considered statistically significant.

## 3. Results and Discussion

### 3.1. PKPs Ameliorate DSS-Induced Colitis Phenotypes in Mice

Given the substantial similarity in pathogenesis between DSS-induced colitis and human UC, we utilized DSS to establish a murine colitis model to evaluate the therapeutic efficacy of PKPs in mitigating disease severity. To holistically assess the therapeutic efficacy of PKPs, we quantified four key colitis-associated phenotypes: body weight change, DAI score, colon length, and splenomegaly. These parameters are established direct indicators of disease progression in the DSS-induced colitis model [15].

**Figure 2:**
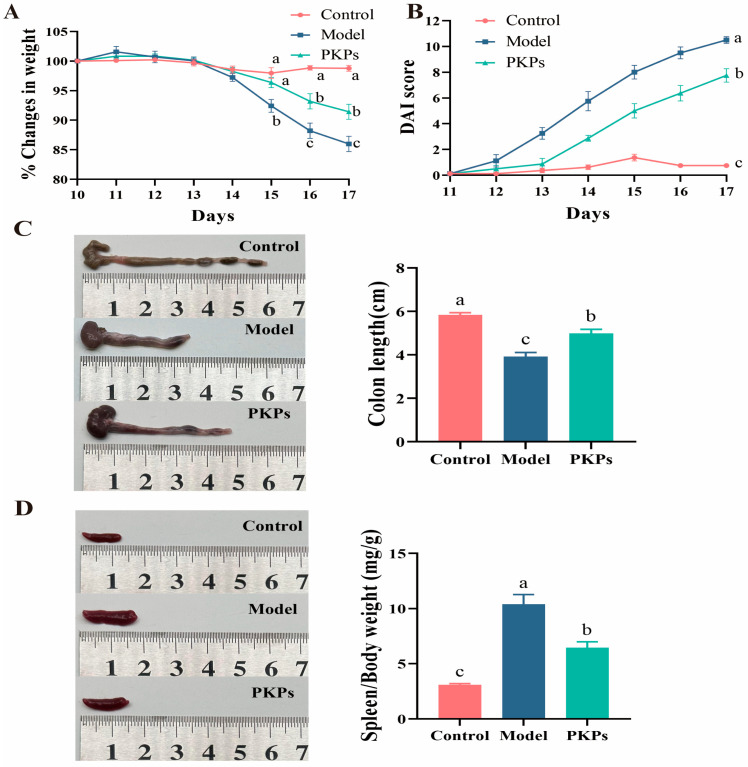
Effects of *Polygonatum kingianum* polysaccharides (PKPs) on the Phenotype of DSS-Induced Colitis in Mice. (**A**) Body weight changes of mice in each group during the experiment. (**B**) DAI scores of mice in each group during the experiment. (**C**) Colon length. (**D**) Spleen index. Data are expressed as mean ± SE (n = 8). For (**A**,**B**), different lowercase letters indicate significant differences among groups at the same time point. Different lowercase letters indicate significant differences (*p* < 0.05).

Results showed that model group mice exhibited significantly lower body weight than the control group on 15th day. Subsequently, by 17th day, PKPs-group mice demonstrated a significant body weight increase (*p* < 0.05) compared to the model group (Figure 2A). Furthermore, mice in the model group developed diarrhea and hematochezia by 13th day. On day 17, PKPs-treated mice exhibited a significantly lower Disease Activity Index (DAI) score relative to the model group (*p* < 0.05; Figure 2B). Colon length in the model group was reduced compared to the control group (*p* < 0.05; Figure 2C). Treatment with PKPs increased colon length in the PKPs group compared to the model group (*p* < 0.05). Furthermore, spleen alterations mirrored those observed in colon length (Figure 2D).

Collectively, these data demonstrate that PKPs significantly alleviate colitis phenotypes, including weight loss, elevated DAI scores, and damage to the colon and spleen, confirming their protective role against DSS-induced colonic pathophysiology.

### 3.2. Effect of PKPs on Intestinal Barrier Function

Intestinal barrier dysfunction, characterized by epithelial damage and tight junction disruption, is a hallmark of ulcerative colitis progression [21]. To determine whether PKPs ameliorate this pathophysiology, we comprehensively assessed colonic histopathology (via H&E staining), tight junction protein expression (ZO-1, Occludin, and Claudin-1), and markers of systemic barrier leakage (DAO, D-lactate, and endotoxin levels) [22].

**Figure 3:**
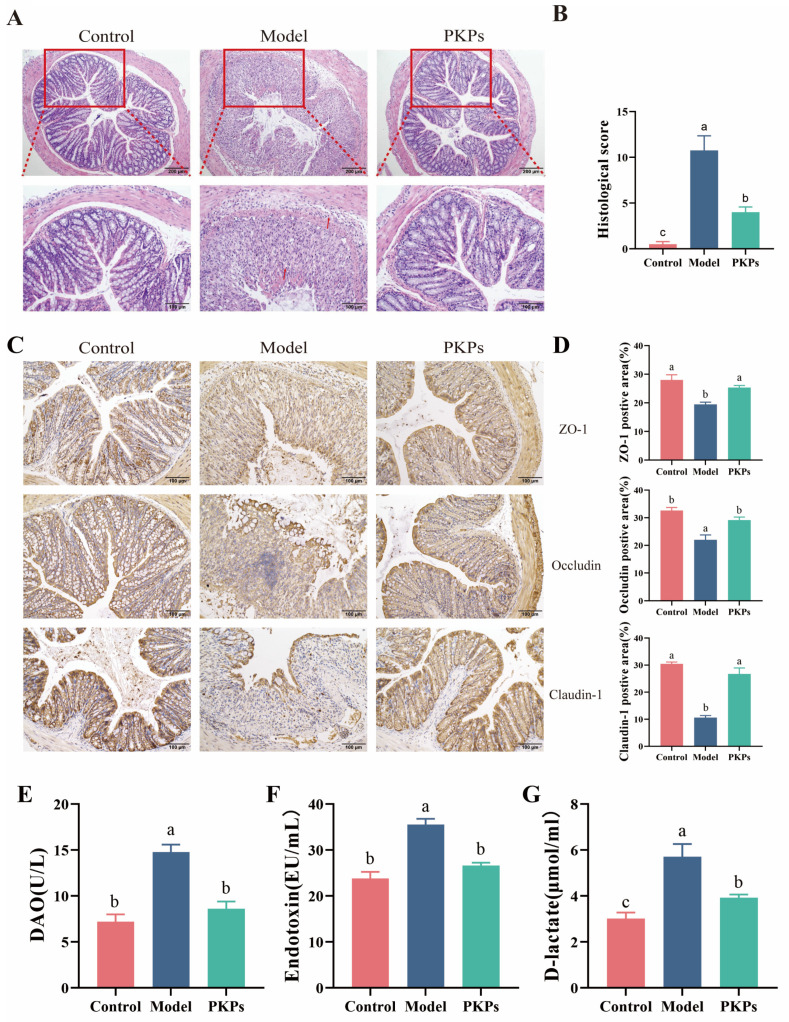
Effects of *Polygonatum kingianum* polysaccharides (PKPs) on Intestinal Barrier Function in DSS-Induced Colitis in Mice. (**A**) H&E staining of colon sections from untreated mice or mice treated with DSS (3%) and PKPs, observed under optical microscopy (magnification: 100× and 200×). The arrows indicate the areas with severe lesions. (**B**) Histological score of colon tissue. (**C**) Descriptive analysis of protein expression of ZO-1, Occludin, and Claudin-1 in colon sections (magnification: 200×). (**D**) Quantification of positive staining area. (**E**) Plasma DAO concentration. (**F**) Plasma endotoxin concentration. (**G**) Plasma D-lactate concentration. Data are expressed as mean ± SE ((**A**–**D**), n = 3; (**E**–**G**), n = 8). Different lowercase letters indicate significant differences (*p* < 0.05).

H&E staining serves as a critical histopathological assessment tool, enabling the detection of subtle pathological alterations in colonic tissues. Histological analysis revealed intact colon tissue architecture in the Control group, characterized by preserved crypt architecture with no apparent inflammatory cell infiltration or tissue damage (Figure 3A). But in the Model group, the colon tissue structure was severely damaged, with disordered or absent crypts, this was associated with notable infiltration of inflammatory cells and mucosal damage, yielding histological scores that significantly exceeded control values by statistical analysis (*p* < 0.05), indicating successful colitis model induction. The PKPs group showed significant improvement in colon tissue structure compared to the Model group, with better crypt alignment, less inflammatory cell infiltration, and reduced mucosal damage. The histological score was markedly lower in comparison to the Model group (*p* < 0.05) (Figure 3B). These results indicate that PKPs exerts protective effects on UC in mice, possibly by reducing inflammatory cell infiltration, repairing crypt structures, and maintaining mucosal barrier function.

Tight junction proteins (TJs) serving as key elements in regulating intestinal epithelial barrier function and tight junction permeability [23]. Previous research has demonstrated a frequent reduction in tight junction protein levels in ulcerative colitis, where these levels are closely correlated with intestinal barrier integrity [21]. TJs, including ZO-1, occludin, and claudin-1, are constituent proteins of the intestinal epithelial barrier [9]. Their function is to regulate paracellular permeability and maintain barrier integrity by forming the tight junctions between epithelial cells [10]. Accumulating evidence indicates that inflammatory mediators alter TJs expression patterns, ultimately compromising gut barrier integrity [15]. In this study, we evaluated the expression of ZO-1, Occludin, and Claudin-1 through immunohistochemical staining, and quantified their expression by analyzing the percentage of positive areas to evaluate intestinal epithelial barrier function. The outcome demonstrated that percentage of positive areas were highly expressed in the Control group (Figure 3C), primarily localized at the membrane region of intestinal epithelial cells, with uniform and continuous staining, indicating intact intestinal barrier function. In the Model group, the expression of these three tight junction proteins were markedly decreased (*p* < 0.05), with discontinuous, absent, and fragmented distribution, and the percentage of positive areas was significantly decreased (Figure 3D), suggesting severe impairment of the colon tissue barrier. In the PKPs group, the expression levels of ZO-1, Occludin, and Claudin-1 were significantly increased (*p* < 0.05), with more positive staining areas and continuous, intact distribution, showing a significant difference than the Model group. These findings indicate that PKPs effectively enhance both the expression and localization of tight junction proteins, aiding in the recovery of compromised intestinal barrier function.

The disruption of intestinal barrier integrity leads to elevated serum DAO, D-Lac, and endotoxin levels [22]. To evaluate the protective effects of PKPs on intestinal barrier homeostasis, we measured these markers. The results (Figure 3E–G) demonstrated that, compared to the Control group, the levels of DAO, D-Lac, and ET were significantly higher in the Model group (*p* < 0.05), respectively, indicating increased intestinal permeability. After PKPs intervention, these markers significantly decreased (*p* < 0.05), respectively, suggesting that PKPs may Contribute positively to alleviating UC by strengthening intestinal barrier integrity.

Overall, through slicing, immunohistochemistry and marker detection, we were able to determine that PKPs prevented the intestinal barrier dysfunction induced by DSS. The alleviation of histopathological damage after PKPs treatment (manifested as the preservation of crypt structures, the reduction in inflammatory infiltration, and the reduction in mucosal ulcers). Crucially, PKPs restored the expression of tight junction proteins (ZO-1 increased, Occluidn increased, Claudin-1 increased), to reconstruct the sealing integrity of intercellular channels. This cellular-level repair directly translated into the overall barrier capacity, which was reflected by the significant decrease in intestinal leakage biomarkers (DAO decrease, D-Lac decrease, ET decrease).

### 3.3. Analysis of Inflammatory Cytokines and Oxidative Dtress Markers in Colon Tissue

DSS-induced colitis is characterized by sustained aberrant immune activation and persistent inflammatory responses within the intestinal mucosa. This inflammatory milieu drives substantial immune cell infiltration and activation, which in turn propels pathogenic cascades. The resultant inflammatory intensity is directly quantifiable through elevated titers of pro-inflammatory cytokines (e.g., TNF-α, IL-6, IL-1β). Concurrently, activated immune cells instigate robust oxidative stress responses, characterized by the generation of reactive oxygen species and redox imbalance. Consequently, quantifying specific inflammatory mediators and oxidative stress biomarkers provides critical functional readouts for assessing the therapeutic efficacy of PKPs in ameliorating experimental colitis.

**Figure 4:**
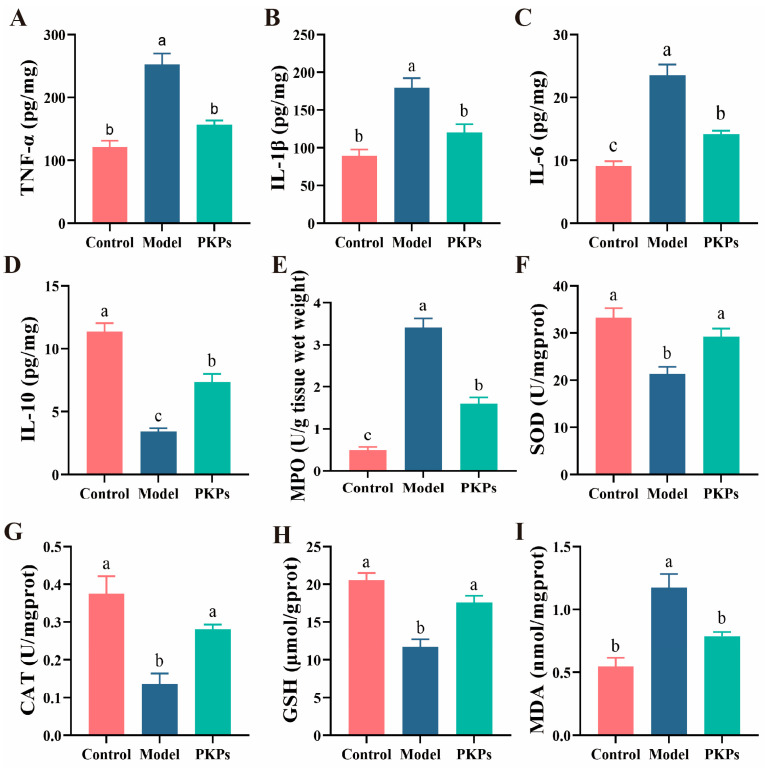
Effects of *Polygonatum kingianum* polysaccharides (PKPs) on Oxidative Stress and Inflammatory Cytokines in Colon Tissue of DSS-Induced Colitis in Mice. (**A**) TNF-α. (**B**) IL-1β. (**C**) IL-6. (**D**) IL-10. (**E**) MPO. (**F**) SOD. (**G**) CAT. (**H**) GSH. (**I**) MDA. Data are expressed as mean ± SE (n = 8). Different lowercase letters indicate significant differences (*p* < 0.05).

This study measured TNF-α, IL-1β, IL-6, and IL-10 (Figure 4A–D). The results showed significant differences in inflammatory cytokine levels between the Control and Model groups (*p* < 0.05). Specifically, the levels of TNF-α, IL-1β, and IL-6 were notably elevated in the Model group in comparison to the Control group, whereas the IL-10 level was significantly reduced in the Model group, indicating that the DSS-induced colitis model successfully triggered a strong inflammatory response. Previous studies have shown that colitis models lead to increased inflammatory cytokines [22], and the secretion of anti-inflammatory cytokines [22]. PKPs intervention significantly improved the levels of cytokines (*p* < 0.05). Specifically, the levels of TNF-α, IL-1β, and IL-6 were markedly lower in the PKPs group (*p* < 0.05), while the IL-10 level was markedly increased, indicating that PKPs potentially mitigate inflammation through dual mechanisms: downregulating pro-inflammatory cytokine secretion while upregulating anti-inflammatory cytokine expression.

MPO activity is associated with oxidative damage and the severity of IBD [24]. In this study, variations in MPO levels further supported the role of PKPs in mitigating the inflammatory response. The MPO levels in the Model group were significantly elevated compared to the Control group (Figure 4E, *p* < 0.05), indicating excessive neutrophil infiltration and inflammation [15]. After PKPs intervention, MPO activity significantly decreased (*p* < 0.05), demonstrating a significant effect in inhibiting the inflammatory response. Subsequently, we evaluated the potential antioxidant properties of PKPs by measuring SOD, CAT, GSH, and MDA levels (Figure 4F–I). Previous studies have shown that DSS-induced colitis mice exhibit reduced levels of CAT, SOD, and GSH, and increased MDA levels, similar to the findings in this study [15]. The Model group showed significantly lower activity of CAT and SOD (*p* < 0.05), indicating impaired antioxidant enzyme system function in the disease model. The significant decrease in GSH levels (*p* < 0.05) further suggested depletion of endogenous antioxidant reserves. Conversely, MDA levels were markedly increased in the Model group (*p* < 0.05), reflecting lipid peroxidation and excessive production of oxidative inflammatory molecules, which exacerbated oxidative damage in colon tissue. However, in the PKPs intervention group, CAT and SOD activity significantly increased (*p* < 0.05), GSH levels significantly recovered (*p* < 0.05), and MDA concentrations significantly reduced (*p* < 0.05), approaching Control group levels.

These findings indicate that PKPs can significantly inhibit lipid peroxidation and the accumulation of inflammatory molecules, improve the oxidative stress status of colon tissue by activating antioxidant enzyme activity and restoring endogenous antioxidant reserves. These findings offer robust support for the potential role of PKPs in alleviating oxidative stress in colitis, and suggest that its mechanism likely mediated through free radical scavenging mechanisms, enhancing antioxidant defense systems, and inhibiting lipid peroxidation. Collectively, these data establish a scientific basis for employing PKPs in the management of oxidative stress-associated pathologies.

### 3.4. Effect of PKPs on the Expression of Nrf2/HO-1/NQO1 Pathway Proteins

In colitis, activated immune cells, particularly neutrophils and macrophages, generate substantial reactive oxygen species (ROS) and reactive nitrogen species (RNS), such as nitric oxide (NO). Concurrently, intestinal antioxidant defenses, encompassing enzymatic components (e.g., superoxide dismutase, SOD) and molecular scavengers (e.g., glutathione, GSH), are frequently impaired. As demonstrated in Section 3.3, this reduction in antioxidant capacity directly correlates with compromised host detoxification of free radicals, resulting in failure to neutralize oxidative stress. Conversely, the Nrf2/HO-1/NQO1 signaling axis represents a critical regulatory mechanism orchestrating cellular antioxidant defenses against oxidative insult.

Current research demonstrates that bioactive polysaccharides from natural sources can potently stimulate Nrf2 activation and subsequent upregulation of its downstream effector genes, leading to significant amelioration of DSS-induced UC pathology [15]. As a transcription factor, Nrf2 controls the expression of downstream antioxidant enzymes HO-1 and NQO1, helping to maintain cellular redox homeostasis and mitigate oxidative stress damage. Study has shown that activation of Nrf2 provides protection in inflammatory diseases like UC by inhibiting oxidative stress, reducing inflammation, and promoting intestinal barrier repair [25].

**Figure 5:**
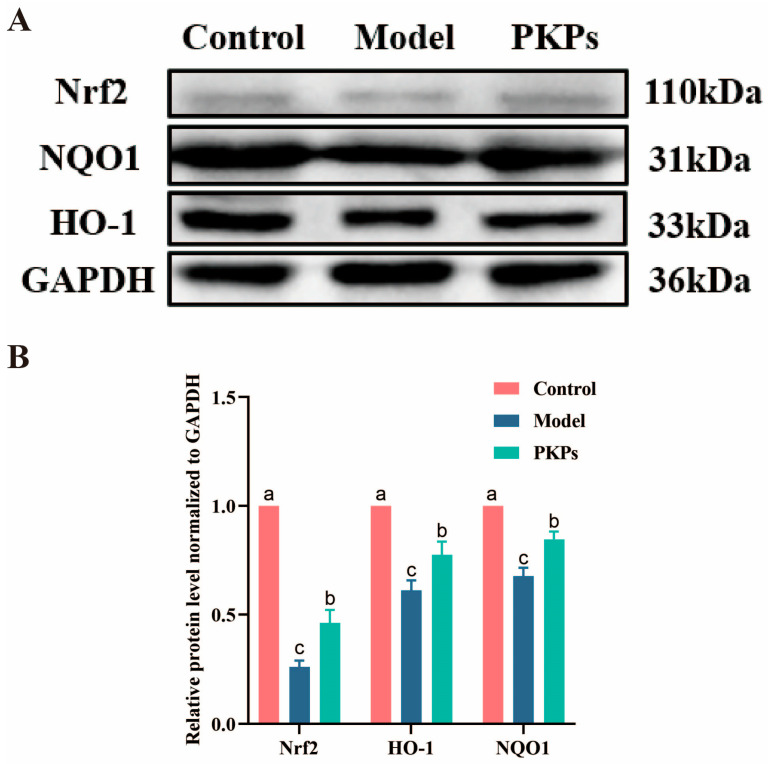
Effects of *Polygonatum kingianum* polysaccharides (PKPs) on the Nrf2, NQO1, and HO-1 Pathways in DSS-Induced Colitis in Mice. (**A**) Western blot analysis of the effects of PKPs on the protein levels of Nrf2, NQO1, and HO-1. (**B**) Protein relative expression normalized to GAPDH. Data are expressed as mean ± SE (n = 4). Different lowercase letters indicate significant differences (*p* < 0.05).

Protein expression levels of Nrf2 and its downstream targets (HO-1 and NQO1) were quantitatively analyzed across experimental groups. Immunoblotting results (Figure 5A,B) revealed markedly higher basal expression of these antioxidant proteins in control animals than model group (*p* < 0.05), suggesting impaired oxidative stress regulation in colitis pathogenesis. Notably, PKPs administration significantly restored Nrf2 pathway activation, with HO-1 and NQO1 expression levels returning to near-normal ranges. These findings demonstrate that PKPs exert potent antioxidant effects in ulcerative colitis through targeted activation of the Nrf2/HO-1/NQO1 signaling axis, effectively counteracting disease-associated oxidative damage.

### 3.5. Effect of PKPs on Gut Microbiota

Given the established centrality of gut microbiota dysbiosis in the pathogenesis of UC and the therapeutic potential of polysaccharides in microbiota modulation, this work systematically characterized PKPs-induced modulations of intestinal microbiota composition and biodiversity in a DSS-induced colitis mouse model [22]. Therefore, this study analyzed the differences in gut microbiota between treatment groups using 16S rRNA sequencing. Utilizing 16S rRNA gene sequencing, we conducted systematic analyses comparing the control, DSS-induced model, and PKPs-treated groups across multiple dimensions: microbial community composition, alpha and beta diversity indices, phylum- and genus-level taxonomic abundance alterations, and shifts in functionally relevant microbial taxa. Our findings demonstrate that PKPs significantly attenuate DSS-induced gut dysbiosis, promote the colonization and recovery of beneficial bacterial populations, and restore a health-oriented microbial ecosystem. The detailed findings regarding gut microbiota analysis are presented below.

**Figure 6:**
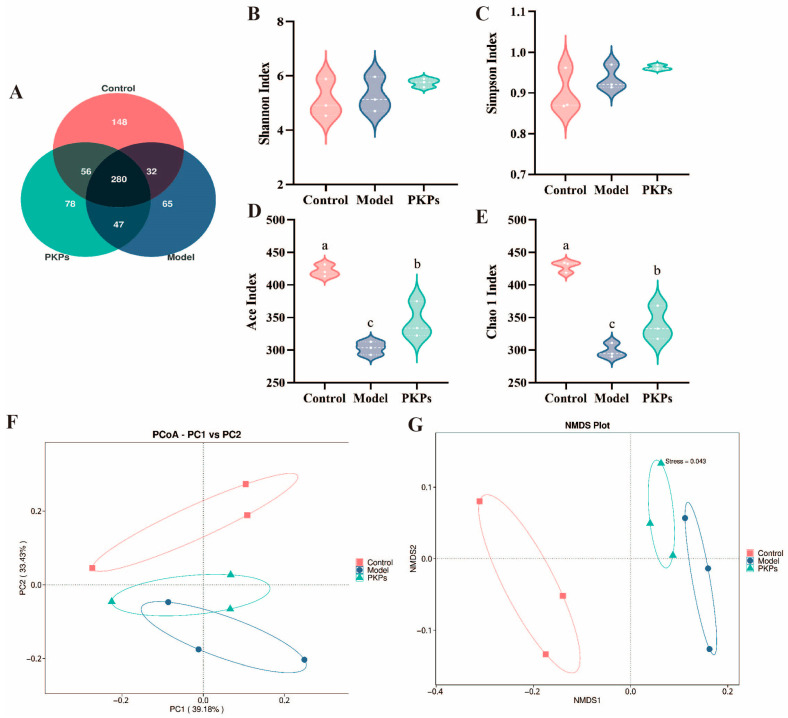
Microbial Diversity Analysis of Intestinal Contents in DSS-Induced Colitis in Mice Treated with *Polygonatum kingianum* polysaccharides (PKPs). (**A**) Venn diagram. (**B**) Shannon Index. (**C**) Simpson Index. (**D**) Ace Index. (**E**) Chao Index. (**F**) PCoA analysis. (**G**) NMDS analysis. Data are expressed as mean ± SE (n = 3). Different lowercase letters indicate significant differences (*p* < 0.05).

The Venn diagram illustrates the shared microbiota communities between the Control, Model, and PKPs groups (Figure 6A). The results showed no significant differences in the number of OTUs among the three groups, with the Control, Model, and PKPs groups containing 516, 424, and 461 OTUs, respectively. Notably, the unique OTUs for each group were 148, 65, and 78, with the PKPs group demonstrating a higher overlap of species with the Control group. These results suggest that PKPs may assist in restoring the composition of the gut microbiota and partially alleviate DSS-induced dysbiosis.

Microbial α-diversity was evaluated through four complementary indices (Figure 6B–E). The species richness estimators (Ace and Chao indices) showed marked variations across experimental groups, consistent with previous findings [26]. In contrast, community diversity metrics (Shannon and Simpson indices) remained relatively stable, indicating preserved microbial evenness despite experimental interventions. This observation aligns with recent reports demonstrating maintained α-diversity under similar conditions [27].

Comparative analysis of microbial richness revealed statistically significant reductions in both Ace and Chao indices for the Model and PKPs groups relative to controls (*p* < 0.05), indicating that DSS induction reduces microbiota richness in mice [9]. However, after PKPs treatment, the richness significantly increased than the Model group (*p* < 0.05), suggesting that PKPs have a beneficial regulatory effect on the diversity of the gut microbiota. PCoA and NMDS analyses of β-diversity showed clear separation in gut microbial profiles between experimental groups [22]. The PCoA and NMDS analysis results (Figure 6F,G) showed that PC1 contributed 39.18%, and PC2 contributed 33.43%. The NMDS stress value was 0.043. These results indicate that through dimensionality reduction analysis, distinct separations were observed in three groups. The Model group’s microbiota was farther from the Control group, suggesting substantial structural changes in the gut microbiota of UC mice. Meanwhile, comparative analysis revealed the PKPs group exhibited microbial composition patterns more closely resembling the Control group, indicating PKPs’ restorative effects on gut microbiota organization.

**Figure 7:**
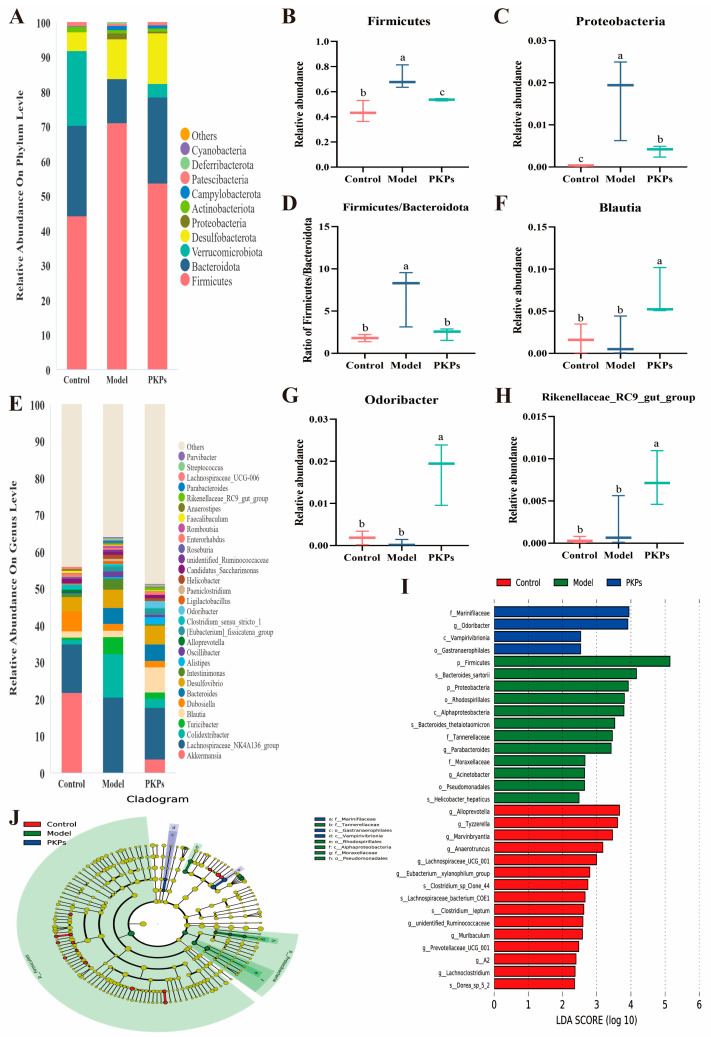
Microbial Community Composition Analysis in Intestinal Contents of DSS-Induced Colitis in Mice Treated with *Polygonatum kingianum* polysaccharides (PKPs). (**A**) Bar plot of the relative abundance of microbial species at the phylum level (top 10 species). (**B**) Firmicutes. (**C**) Proteobacteria. (**D**) Firmicutes/Bacteroidota ratio. (**E**) Bar plot of the relative abundance of microbial species at the genus level (top 30 species). (**F**) Blautia. (**G**) Odoribacter. (**H**) Rikenellaceae_RC9_gut_group. (**I**) LefSe analysis LDA score plot. (**J**) LefSe analysis taxonomic branching plot. Data are expressed as mean ± SE (n = 3). Different lowercase letters indicate significant differences (*p* < 0.05).

There were no significant differences in the relative abundance of microbiota at the phylum level across the groups (Figure 7A), with *Firmicutes*, *Bacteroidota*, *Verrucomicrobiota*, *Desulfobacterota*, *Proteobacteria*, *Actinobacteriota*, *Campylobacterota*, *Patescibacteria*, *Deferribacterota*, and *Cyanobacteria* comprising over 99% of the microbiota in each group. Notably, following DSS treatment, the relative abundances of *Firmicutes* and *Proteobacteria* were elevated in the Model group (*p* < 0.05), with increases of 60.73% and 53.46%, respectively, compared to the Control group (Figure 7B,C). After PKPs treatment, these two phyla were significantly downregulated by 24.46% and 77.45%, respectively, compared to the Model group (*p* < 0.05). Previous studies have indicated that colitis results in an increase in *Firmicutes* and *Proteobacteria* [28,29]. Interestingly, decreased *Firmicutes* abundance has been reported in colitis models, contrasting with our observations, which contrasts with our findings. The changes in *Firmicutes* abundance continue to be a subject of debate [28]. It has been suggested that an increased *Firmicutes*/*Bacteroidota* (F/B) ratio contributes to the initiation and progression of UC [30]. In this study, the F/B ratio was 1.80 ± 0.25, 6.99 ± 1.96, and 2.32 ± 0.41 for the Control, Model, and PKPs groups, respectively. The Model group exhibited a value approximately 4-fold higher than the Control group (Figure 7D) and about 3-fold higher than the PKPs group, indicating severe inflammation after DSS induction and marked alleviation following PKPs treatment. *Proteobacteria* is commonly used as an indicator of dysbiosis in the gut microbiota, with increased abundance potentially indicating the presence of pathogenic bacteria [31]. For *Verrucomicrobiota*, its abundance is significantly reduced in UC patients [32], and similarly, DSS induction leads to a reduction in *Verrucomicrobiota* abundance in mice [33]. In our study, The Model group exhibited a significant reduction (*p* < 0.05), while the PKPs group showed a trend of recovery, suggesting that PKPs may promote the growth of *Verrucomicrobiota*, enhancing gut ecological balance.

At the genus level, the top 30 relative abundances of microbiota were analyzed (Figure 7E). The main genera included Akkermansia, Lachnospiraceae_NK4A136_ group, Colidextribacter, Turicibacter, Blautia, Dubosiella, Bacteroides, Desulfovibrio, Intestinimonas, and Alistipes. Notably, the PKPs group demonstrated a marked elevation in the relative abundance of three key bacterial taxa: Blautia, Odoribacter, and Rikenellaceae_RC9_gut_group (Figure 7F–H). The abundance of Blautia in the PKPs group was approximately three times higher than in other groups. Blautia enrichment correlates with enhanced lipid homeostasis and upregulated tight junction protein (ZO-1/Occludin) expression, as reported in prior research [34]. Blautia is also considered a potential probiotic for regulating gut microbiota and could be an effective treatment for preventing and treating colitis [35]. The abundance of Odoribacter in the PKPs group was significantly higher than that in both the Control and Model groups (p < 0.05). Odoribacter is a SCFAs producer, especially butyrate [36], and is also related to insulin regulation [37]. Research has indicated that oral delivery of Odoribacter laneus improves glucose homeostasis and alleviates inflammation in obese mice [38]. Odoribacter splanchnicus has been shown to respond to fecal microbiota transplantation to treat UC and limit colon inflammation [39]. Furthermore, the metabolites of Odoribacter can promote colonic mucosal healing in colitis [40]. The abundance of Rikenellaceae_RC9_gut_group in the PKPs group was higher than that in both the Control and Model groups (p < 0.05).We speculate that this genus might be passively enriched during colitis as a compensatory response to gut microbiota dysbiosis. It may temporarily expand in an impaired intestinal barrier or inflammatory environment to help regulate immune responses or maintain local homeostasis. After PKPs treatment, the secretion of Rikenellaceae_RC9_gut_group accelerated, alleviating colon inflammation. Rikenellaceae_RC9_gut_group, a member of Bacteroidetes, is a bacterium that produces butyrate [41] and is associated with a high-fiber diet that shields mice from DSS-induced UC [42].

LEfSe analysis (Figure 7I,J) was employed to identify the dominant microbial taxa at multiple taxonomic levels among the experimental groups. The Control group was characterized by the enrichment of *g_Alloprevotella*, *g_Tyzzerella*, *g_Marvinbryantia*, and *g_Anaerotruncus*. In contrast, the Model group exhibited higher relative abundances of *p_Firmicutes*, *s_Bacteroides sartorii*, *p_Proteobacteria*, and *o_Rhodospirillales*. Notably, the PKPs group showed a predominance of beneficial taxa, including *f_Marinifilaceae*, *g_Odoribacter*, *c_Vampirivibrionia*, and *o_Gastranaerophilales*. Previous studies have highlighted the association of *s_Bacteroides* sartorii with dysbiosis in diarrheic mice [43] and reported increased abundances of *g_Bacteroides* and *p_Proteobacteria* in models simultaneously exposed to DSS and high-fructose diets [44]. Additionally, human studies have linked *o_Rhodospirillales* enrichment to disease progression in multiple sclerosis [45], further supporting the observed dysbiosis in the Model group. Conversely, *f_Marinifilaceae* has been reported to alleviate lipid metabolism disorders [46], while reductions in the relative abundances of *c_Vampirivibrionia* in IBD-affected dogs [47] and *o_Gastranaerophilales* in high-fat, high-sugar-induced mouse models [42] underscore their potential protective roles.

In conclusion, PKPs appear to regulate the gut microbiota in DSS-induced colitis mice. PKPs enhance the abundance of *Marinifilaceae*, *Vampirivibrionia*, *Gastranaerophilales*, *Blautia*, *Odoribacter*, *Rikenellaceae_RC9_ gut_group*, enhancing the diversity of the gut microbiota and reestablishing the balance of the intestinal microecology, thus promoting gut barrier function and immune responses.

### 3.6. Effect of PKPs on SCFAs in Feces

**Figure 8:**
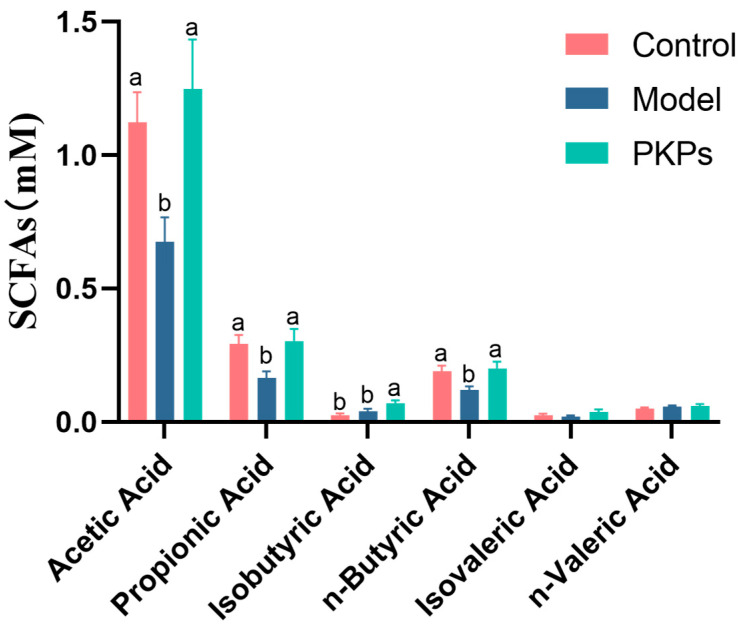
Effects of *Polygonatum kingianum* polysaccharides (PKPs) on Short-Chain Fatty Acids in Intestinal Contents of DSS-Induced Colitis in Mice. Data are expressed as mean ± SE (n = 8). Different lowercase letters indicate significant differences (*p* < 0.05).

Building upon our findings demonstrating PKPs’ significant modulation of gut microbiota structure, and considering the pivotal role of short-chain fatty acids (SCFAs)—key microbial metabolites—in maintaining host immune homeostasis and colonic health [48], this study further quantified fecal levels of major SCFAs across experimental groups (Figure 8). Study has shown that SCFAs influence immune cell differentiation and the gut microbiota composition in mice with colitis [48]. Through short-chain fatty acid metabolic data (Figure 8), we observed that the levels of acetate, propionate, and butyrate in the Model group were significantly lower than in the Control group (*p* < 0.05), which is consistent with the typical features of colitis [48]. However, in the PKPs-treated group, after PKPs intervention, the levels of these three SCFAs significantly restored to normal levels (*p* > 0.05), suggesting that PKPs may help reestablish the balance of gut microbiota and alleviate pathological changes in colitis by regulating gut metabolites.

Moreover, no significant variations were observed in the levels of isovaleric acid and valeric acid across the groups. This implies that PKPs may have a limited impact on the metabolism of these SCFAs, which aligns with previous studies [49]. Notably, the level of isobutyric acid was significantly elevated in the PKPs-treated group in comparison to both the Control and Model groups (*p* < 0.05). This result suggests that PKPs may exert anti-inflammatory and gut-repairing effects by promoting the synthesis or absorption of isobutyric acid. Previous research has also shown that the level of isobutyric acid in positive control groups significantly increases [50]. SCFAs are associated with the high abundance of genera such as *Blautia* [51], and *Rikenellaceae_RC9_gut_group* [41]. Therefore, we hypothesize that PKPs alleviate symptoms of UC by promoting the growth of genera such as *Blautia* and restoring the levels of SCFAs, particularly isobutyric acid. This finding is consistent with multiple studies indicating that SCFAs, particularly acetate, propionate, and butyrate, exert anti-inflammatory effects by modulating gut immune responses, promoting intestinal barrier function, and maintaining gut microecological balance [49].

### 3.7. Untargeted Metabolomics Data Analysis of PKPs

**Figure 9:**
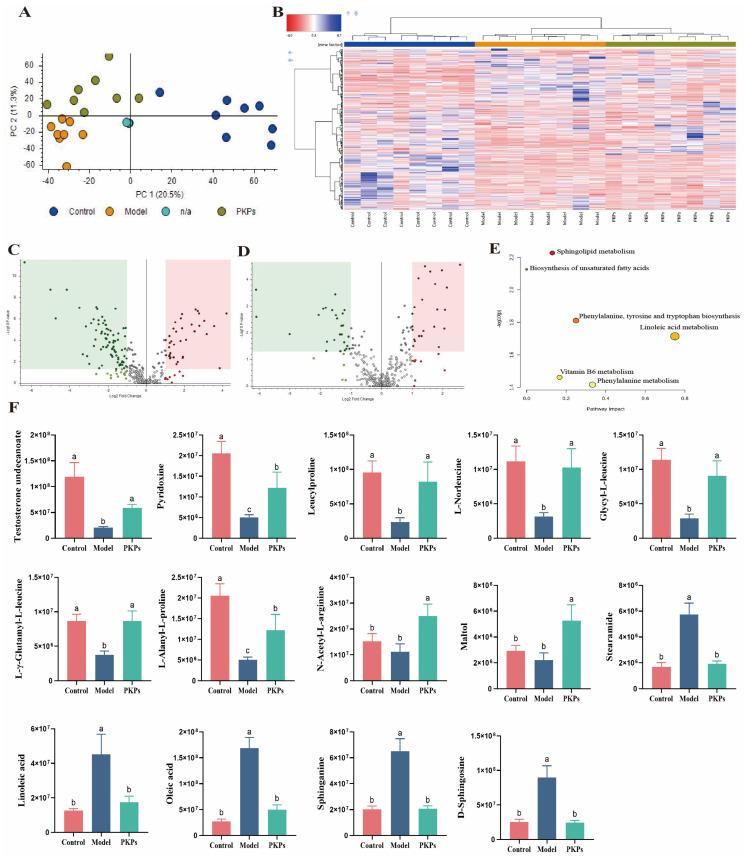
Effects of *Polygonatum kingianum* polysaccharides (PKPs) on Metabolites in Feces of DSS-Induced Colitis in Mice. (**A**) PCA plot. (**B**) Cluster analysis heatmap. (**C**) Volcano plot of differential metabolites between Model and Control. (**D**) Volcano plot of differential metabolites between Model and PKPs. No color is used to represent upregulated and downregulated compounds. (**E**) Metabolic pathways. (**F**) Differential metabolites among the three groups. Data are expressed as mean ± SE (n = 8). Different lowercase letters indicate significant differences (*p* < 0.05).

To elucidate the microbiota-host interactions and PKPs-mediated colitis amelioration, this study performed fecal metabolomic profiling across experimental groups using UHPLC-MS (Figure 9). Comprehensive metabolomic analytical outcomes are presented below.

In positive ion mode, a total of 6609 molecular features were detected across all samples. In this study, we performed principal component analysis (PCA) on the metabolomics data to assess the overall differences in metabolic characteristics. In the PCA plot (Figure 9A), we observed that QC samples clustered tightly together, indicating stable QC samples and high reproducibility of the metabolomics method and instrument stability. The Control, Model, and PKPs groups also clustered together within their respective groups, separating from each other, indicating consistent metabolic variation and phenotypic differences. Meanwhile, a clear distinction among the three groups was evident, with PC1 and PC2 contributing 20.5% and 11.3% to the separation, respectively, explaining a total of 31.8% of the overall metabolic differences. Heatmap clustering analysis was then used to assess the trends of metabolites across the groups (Figure 9B). We observed significant metabolic changes in the feces of DSS-induced mice, and after PKPs treatment, the metabolites in the mice’s feces were partially restored, suggesting that PKPs have a mitigating effect on DSS-induced UC.

To identify the specific potential metabolites involved in PKPs-mediated relief of colitis, we conducted differential metabolite screening. We identified 80 downregulated and 37 upregulated metabolites in the Model group in comparison to the Control group, with 117 common differential metabolites between the two groups (Figure 9C). Compared to the PKPs group, the Model group exhibited 27 downregulated and 23 upregulated metabolites, with 50 common differential metabolites between the two groups (Figure 9D). We defined metabolites that showed opposite trends after DSS exposure and PKPs supplementation as potential biomarker metabolites, identifying 35 differential metabolites (Table S3).

In comparison to the Control group, 15 substances, including Testosterone undecanoate, Pyridoxine, Leucylproline, L-Norleucine, Glycyl-L-leucine, Leucylphenylalanine, L-γ-Glutamyl-L-leucine, L-Alanyl-L-proline, etc., were markedly decreased in the Model group. After PKPs treatment, these metabolites showed significant upregulation and were brought back to near-normal levels. Additionally, after DSS treatment, 15 substances, including Linoleic acid, Oleic acid, Stearamide, Sphinganine, and D-Sphingosine, were enriched in the Model group, but were significantly downregulated after PKPs treatment. Interestingly, although differences were not statistically significant in Maltol, N-α-L-Acetyl-arginine, and Docosahexaenoic acid ethyl ester in the Control and Model groups, these metabolites showed opposite significant trends in the PKPs group compared to the Model group. We then used the KEGG database in MetaboAnalyst 4.0 for enrichment analysis of the altered metabolites to identify metabolic pathways that PKPs may be involved in. Four major metabolic pathways were identified: “Sphingolipid metabolism,” “Biosynthesis of unsaturated fatty acids,” “Phenylalanine, tyrosine, and tryptophan biosynthesis,” and “Linoleic acid metabolism” (Figure 9E).

Some key differential metabolites were further analyzed (Figure 9F). In a human study, testosterone undecanoate levels were found to be altered in male patients with Crohn’s disease-induced hypogonadism, and supplementation with testosterone undecanoate showed positive effects on the disease [52]. Pyridoxine is an essential nutrient required for the normal functioning of many biological systems, and a previous study has shown that reduced levels of pyridoxine are common in IBD patients, with similar findings in this study [53]. UC leads to a decrease in amino acids and their derivatives [54], and among the key metabolites identified in this study, Leucylproline has been suggested as a key metabolite for treating irritable bowel syndrome [55]. Research has indicated that Leucylproline levels are reduced in patients with colon cancer [56]. L-Norleucine, an isomer of leucine, has plasma concentrations inversely associated with colorectal cancer [57]. L-γ-Glutamyl-L-leucine reflects a higher antioxidant state and nutritional metabolism in the body [58]. In the Rotenone-induced Parkinson’s disease model, L-Alanyl-L-proline levels were found to decrease [59]. By increasing Glutamyl-L-leucine levels, it alleviates liver injury with respect to the gut-liver axis [60]. N-α-L-Acetyl-arginine plays a fundamental role as a metabolic precursor in the arginine biosynthesis pathway and is widely regarded as an indicator of normal cellular metabolic activity. Current research indicates that arginine exerts beneficial effects on gastrointestinal health by promoting the maintenance of intestinal epithelial barrier function and reducing the incidence of colitis [61]. Maltol possesses antioxidant and anti-inflammatory activities that confer beneficial effects on IBD [62]. Study has shown that Stearamide is significantly elevated in individuals with irritable bowel syndrome comorbid with depression [63]. In this study, PKPs treatment also reduced Stearamide levels.

In conclusion, it is speculated that PKPs alleviate UC by restoring the levels of the aforementioned metabolites with therapeutic potential. Research has indicated that colitis leads to the accumulation of the saturated and unsaturated fatty acids [64]. In our study, PKPs normalized the elevated levels of oleic acid and linoleic acid in the feces of mice, aligning with findings from previous research [64,65]. Linoleic acid is a mixture of positional (e.g., 7, 9; 9, 11; 10, 12; 11, 13) and geometric (cis or trans) isomers. Linoleic acid accumulation suppresses Treg cell differentiation through macrophage infiltration and increased pro-inflammatory cytokine production, thus heightening colitis susceptibility [66]. Furthermore, a high linoleic acid diet increases colitis susceptibility in mice, and reducing Linoleic acid levels alleviates colitis [67]. These findings further confirm that PKPs influence the metabolic pathways “Biosynthesis of Unsaturated Fatty Acids” and “Linoleic Acid Metabolism,” positively impacting UC. Sphingolipids are involved in IBD, possibly affecting mucosal integrity, barrier function, and receptor activity, as well as the generation of sphingolipid signaling molecules in epithelial and inflammatory cells [68]. The differential metabolites Sphinganine and D-Sphingosine identified in this study further confirm that PKPs positively influence UC by affecting the “Sphingolipid Metabolism” pathway.

### 3.8. Combined Analysis of Gut Microbiota, Biochemical Indicators, and Metabolites

**Figure 10:**
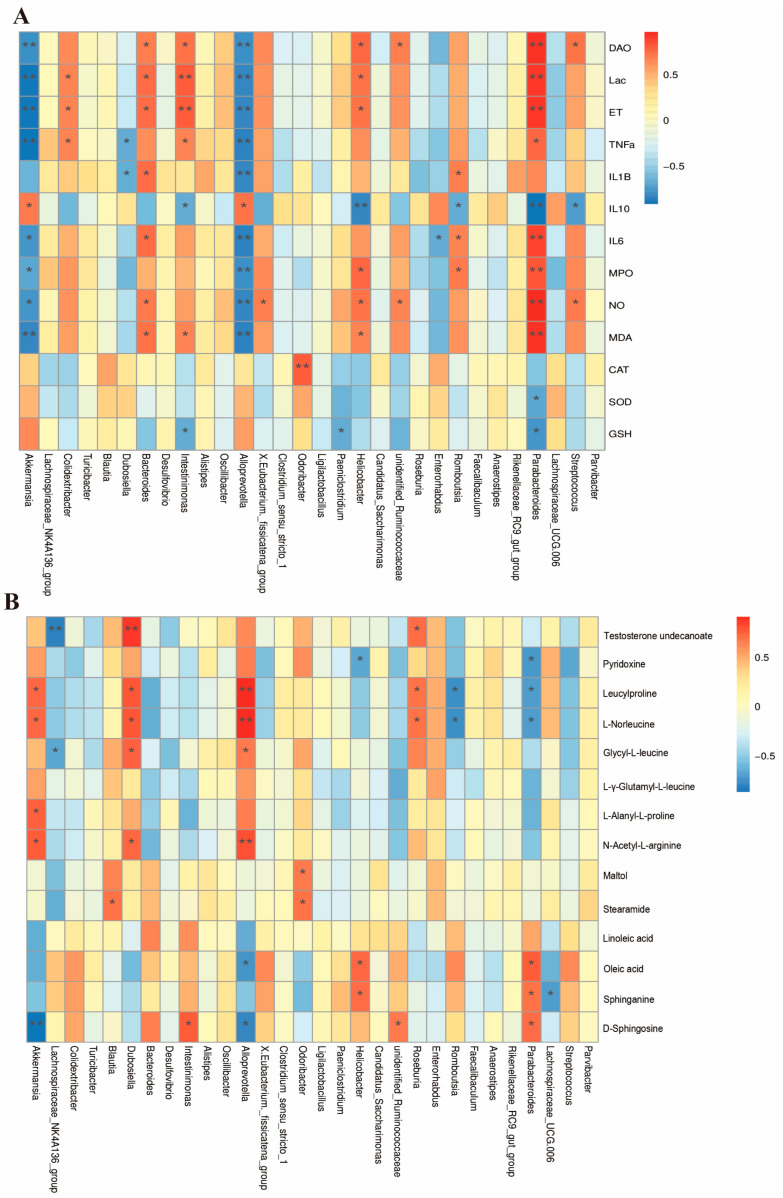
Spearman Correlation Heatmap of DSS-Induced Colitis in Mice Treated with *Polygonatum kingianum* polysaccharides (PKPs). (**A**) Correlation analysis between gut microbiota and biochemical indices. (**B**) Correlation analysis between gut microbiota and differential metabolites. * indicate significant differences (*p* < 0.05), ** indicate significant differences (*p* < 0.01).

To systematically deconvolve the synergistic network underlying PKPs-mediated colitis alleviation, we constructed a tripartite evidence framework integrating gut microbiota, metabolomic profiles, and host biochemical indicators. Key bacterial genera-metabolite-inflammatory marker interactions were assessed using Spearman’s rank correlation. (Figure 10). This integrative approach elucidates PKPs’ mechanism in restoring microbiota-metabolite-host homeostasis at the ecological network level. Detailed multi-omics integration findings follow below.

To thoroughly investigate the relationships among fecal metabolites, gut microbiota, and biochemical markers, we conducted Spearman correlation analyses between these variables. The association analysis focused on the top 30 most abundant genera of gut microbiota and biochemical indicators (Figure 10A), we found significant negative correlations (*p* < 0.05) between DAO, Lac, ET, TNF-α, IL-1β, IL-6, MPO, NO, MDA and *Akkermansia*, *Dubosiella*, *Alloprevotella*, and *Dubosiella*. These were significantly positively correlated (*p* < 0.05) with *Colidextribacter*, *Bacteroides*, *Intestinimonas*, *Romboutsia*, *Helicobacter*, *Streptococcus*, and *Parabacteroides*.

Through the association analysis of gut microbiota at the genus level (top 30 abundances) and fecal metabolites (Figure 10B), we observed that *Akkermansia*, *Dubosiella*, *Alloprevotella*, and *Roseburia* were positively correlated with amino acid-related metabolites (e.g., Leucylproline, L-Norleucine, N-Acetyl-L-arginine) and negatively correlated with Oleic acid and D-Sphingosine. *Lachnospiraceae_NK4A136_group* was negatively correlated with Testosterone undecanoate and Glycyl-L-leucine, while *Romboutsia* and *Helicobacter* were inversely associated with Pyridoxine, Leucylproline, and L-Norleucine, and positively correlated with Oleic acid, Sphinganine, and D-Sphingosine. These results suggest that PKPs may modulate specific gut microbiota, thereby influencing multiple metabolic pathways, playing a synergistic role in anti-inflammatory effects and mucosal repair.

## 4. Conclusions

PKPs exert protective effects in a DSS-induced murine colitis model. PKPs alleviate key symptoms like weight loss, colon shortening, and splenomegaly while promoting intestinal mucosal healing. PKPs exert immunomodulatory effects through suppression of pro-inflammatory cytokines (TNF-α, IL-1β, IL-6) and enhancement of anti-inflammatory IL-10. They also enhance antioxidant defense via the Nrf2/HO-1/NQO1 pathway. Additionally, PKPs modulate gut microbiota by enriching beneficial bacteria and restoring SCFA levels, alongside regulating metabolic pathways. These findings suggest PKPs mitigate colitis through multi-target mechanisms involving immune regulation, oxidative stress reduction, microbiota modulation, and metabolic adjustment. In future work, more comprehensive structural characterization and systematic dose–response studies of PKPs will be conducted to further optimize their therapeutic potential and application prospects.

## Data Availability

The original contributions presented in this study are included in the article/Supplementary Materials. Further inquiries can be directed to the corresponding authors.

## Supplementary Materials

The following supporting information can be downloaded at: https://www.mdpi.com/article/10.3390/nu17172895/s1, Material S1. Immunohistochemistry. Material S2. Western blot assay. Material S3. Sample size justification. Table S1. Disease Activity Index (DAI) scoring criteria for DSS-induced acute ulcerative colitis in mice. Table S2. Histologic scoring criteria for DSS-induced acute ulcerative colitis in mice. Table S3. Potential biomarkers after DSS exposure and PKPs supplementation.

## Abbreviations

The following abbreviations are used in this manuscript:

PKPs	*Polygonatum kingianum* Collett & Hemsl Polysaccharides
DSS	Dextran sulfate sodium
UC	Ulcerative colitis
SPF	Specific Pathogen–Free
DAO	Diamine Oxidase
D-Lac	D-lactate
ET	Endotoxin
MPO	Myeloperoxidase
SOD	Superoxide Dismutase
MDA	Malondialdehyde
CAT	Catalase
GSH	Glutathione
TJs	Tight junction proteins
IBD	Inflammatory bowel diseases
PCA	Principal component analysis
UPLC-MS	untargeted ultra-performance liquid chromatography-mass spectrometry
