# Supplementary material for: Integrative Multi-Omics Reveals the Anti-Colitis Mechanisms of Polygonatum kingianum Collett & Hemsl Polysaccharides in a Mouse DSS Model
Source: Nutrients. 2025 Sep 8;17(17):2895. doi: 10.3390/nu17172895 (PMC12430059; doi:10.3390/nu17172895)
Supplement: Supplementary file 1 [file nutrients-17-02895-s001.zip › nutrients-3788536-supplementary.pdf]

Supplementary Material S1. Immunohistochemistry.

Immunohistochemical analysis was performed to assess the expression of ZO-1, Occludin, and Claudin-1 in colon tissues. After deparaffinization, rehydration, washing, antigen retrieval, and endogenous peroxidase removal, tissue sections were incubated in PBS containing 3% bovine serum albumin (BSA) for 30 minutes. The sections were then incubated with primary antibodies (Occludin, Claudin-1, and ZO-1) provided by Servicebio, Wuhan, China, at 4°C for 12 hours. After primary antibody incubation, sections were washed thoroughly and incubated with corresponding secondary antibodies at room temperature (25°C) for 50 minutes.Following incubation, the sections were washed again and stained with DAB.

Supplementary Material S2. Western blot assay.

Each colon sample was weighed and homogenized in lysis buffer containing protease and phosphatase inhibitors at a ratio of 1:9 (w/v). The tissue homogenate was centrifuged at 10,000g for 5 minutes at 4°C, and the supernatant was collected. Protein concentration was determined, and 30μg of protein was loaded onto a 10% SDS-PAGE gel. After electrophoresis, the target protein was transferred to a polyvinylidene fluoride (PVDF) membrane. The membrane was incubated with primary antibodies overnight at 4°C, washed, and then incubated with secondary antibodies for 1 hour at room temperature. The protein expression was detected using ECL chemiluminescence reagents.

Supplementary Material S3. Sample size justification

In this study, sample sizes varied according to the characteristics of each assay and followed common practices reported in the literature. For macroscopic evaluations, plasma markers, and SCFAs, n=8 per group was used due to higher inter-animal variability, ensuring stable statistical results while adhering to the 3R principle. For Western blot, n=4 per group was chosen because protein expression typically shows lower variability but requires considerable sample material and antibodies; this number balances material use with statistical robustness. For immunohistochemistry and microbiota analysis, n=3 per group was sufficient, as each sample yields multiple independent data points (e.g., non-overlapping microscopic fields or sequencing reads). This approach is widely adopted in DSS-colitis studies. In summary, the different n values used across assays were determined based on methodological considerations, ethical principles, and consistency with previously published studies.

Table S1. Disease Activity Index (DAI) scoring criteria for DSS-induced acute ulcerative colitis in mice

DAI score	Weight loss (%)	Fecal hardness	Fecal occult blood
0	0	Normal	Normal
1	1-4	Moderately loose feces	Presence of blood
2	5-9	Loose stools	Moderate bleeding
3	10-19	Loose stool, diarrhea	Moderately high
4	>20	Diarrhea	Abundant bleeding

Table S2. Histologic scoring criteria for DSS-induced acute ulcerative colitis in mice.

Score	Inflammation	Mucosal damag	Crypt damage	Rang of lesions
0	None	None	None	0
1	Mild	Mucous layer	1/3	1-25
2	Moderate	Submucosa	2/3	26-50
3	Severe	Muscularis and serosa	100%	51-75
4	-	-	100%+epithelium loss	76-100

Table S3. Potential biomarkers after DSS exposure and PKPs supplementation.

PubChem ID	Name	Formula	M/C	M/P	P/C
80817	Leucylproline	C11 H20 N2 O3	↓	↑	n.s.
65157	Testosterone undecanoate	C30 H48 O3	↓	↑	n.s.
9837272	Valylproline	C10 H18 N2 O3	↓	↑	n.s.
10828227	2,3-dihydroxypropyl 12-methyltridecanoate	C17 H34 O4	↓	↑	↓
92843	Glycyl-L-leucine	C8 H16 N2 O3	↓	↑	n.s.
144746	N-Valylphenylalanine	C14 H20 N2 O3	↓	↑	n.s.
114829	4',7-Dihydroxyflavanone	C15 H12 O4	↓	↑	n.s.
6992310	Leucylphenylalanine	C15 H22 N2 O3	↓	↑	n.s.
21236	L-Norleucine	C6 H13 N O2	↓	↑	n.s.
151023	L-γ-Glutamyl-L-leucine	C11 H20 N2 O5	↓	↑	n.s.
637542	p-coumaric acid	C9 H8 O3	↓	↑	n.s.
997	Phenylpyruvic acid	C9 H8 O3	↓	↑	n.s.
444539	3-Phenylacrylic acid	C9 H8 O2	↓	↑	n.s.
1054	Pyridoxine	C8 H11 N O3	↓	↑	↓
83525	L-Alanyl-L-proline	C8 H14 N2 O3	↓	↑	↓
8369	Maltol	C6 H6 O3	n.s.	↑	n.s.
67427	N-α-L-Acetyl-arginine	C8 H16 N4 O3	n.s.	↑	n.s.
9831416	Docosahexaenoic acid ethyl ester	C24 H36 O2	n.s.	↓	n.s.
656816	Hexadecasphinganine	C16 H35 N O2	↑	↓	n.s.
2802958	2-Aminooctadec-4-yne-1,3-diol	C18 H35 N O2	↑	↓	↑
3931	Linoleic acid	C18 H32 O2	↑	↓	n.s.
10631	Medroxyprogesterone	C22 H32 O3	↑	↓	n.s.
31292	Stearamide	C18 H37 N O	↑	↓	n.s.

12921	Sterculic acid	C19 H34 O2	↑	↓	n.s.
1711945	Farnesylacetone	C18 H30 O	↑	↓	n.s.
91486	Sphinganine	C18 H39 N O2	↑	↓	n.s.
9860720	2-amino-2-tetradecylpropane-1,3-diol	C17 H37 N O2	↑	↓	n.s.
10958200	cis-5,8,11-Eicosatrienoic acid methyl ester	C21 H36 O2	↑	↓	n.s.
5280335	D-Sphingosine	C18 H37 N O2	↑	↓	n.s.
5281852	5-(8-Pentadecenyl)-1,3-benzenediol	C21 H34 O2	↑	↓	n.s.
445639	Oleic acid	C18 H34 O2	↑	↓	n.s.
247839	Medrysone	C22 H32 O3	↑	↓	n.s.
12963317	cis-12-Octadecenoic acid methyl ester	C19 H36 O2	↑	↓	↑

↑ represents that the metabolite is up-regulated in the comparison of the two groups. ↓ represents that the metabolite is down-regulated in the comparison of the two groups. n.s. represents no significant difference between the two groups.
